# Quantitative label-free proteomic analysis of excretory-secretory proteins in different developmental stages of *Trichinella spiralis*

**DOI:** 10.1186/s13567-023-01258-7

**Published:** 2024-01-03

**Authors:** Yadong Liu, Juncheng Liu, Nan Wang, Xihuo You, Yaming Yang, Jing Ding, Xiaolei Liu, Mingyuan Liu, Chen Li, Ning Xu

**Affiliations:** 1 State Key Laboratory for Diagnosis and Treatment of Severe Zoonotic Infectious Diseases, Key Laboratory for Zoonosis Research of the Ministry of Education, Institute of Zoonosis, and College of Veterinary Medicine, Jilin University, Changchun, 130062 China; 2 College of Veterinary Medicine, Shandong Agricultural University, Tai’an, 271018 China; 3 Jilin Agricultural University, Changchun, 130062 China; 4 Beijing Agrichina Pharmaceutical Co., Ltd., Wangzhuang Industrial Park, Airport Road, Shahe, Changping District, Beijing, 102206 China; 5 Yunnan Institute of Parasitic Diseases, 6 Xiyuan Road, Puer, Yunnan China

**Keywords:** *Trichinella spiralis*, excretory-secretory proteins, label-free, proteomics, LC‒MS/MS

## Abstract

*Trichinella spiralis* (*T. spiralis*) is a zoonotic parasitic nematode with a unique life cycle, as all developmental stages are contained within a single host. Excretory-secretory (ES) proteins are the main targets of the interactions between *T. spiralis* and the host at different stages of development and are essential for parasite survival. However, the ES protein profiles of *T. spiralis* at different developmental stages have not been characterized. The proteomes of ES proteins from different developmental stages, namely, muscle larvae (ML), intestinal infective larvae (IIL), preadult (PA) 6 h, PA 30 h, adult (Ad) 3 days post-infection (dpi) and Ad 6 dpi, were characterized via label-free mass spectrometry analysis in combination with bioinformatics. A total of 1217 proteins were identified from 9341 unique peptides in all developmental stages, 590 of which were quantified and differentially expressed. GO classification and KEGG pathway analysis revealed that these proteins were important for the growth of the larvae and involved in energy metabolism. Moreover, the heat shock cognate 71 kDa protein was the centre of protein interactions at different developmental stages. The results of this study provide comprehensive proteomic data on ES proteins and reveal that these ES proteins were differentially expressed at different developmental stages. Differential proteins are associated with parasite survival and the host immune response and may be potential early diagnostic antigen or antiparasitic vaccine candidates.

**Supplementary Information:**

The online version contains supplementary material available at 10.1186/s13567-023-01258-7.

## Introduction

*Trichinella spiralis* is an intracellular parasite. First discovered in 1835, it is highly prevalent in domestic and sylvatic animals. *T. spiralis* infects a wide range of carnivores and omnivores and is the main pathogenic agent of human trichinellosis [1]. Humans become infected via the consumption of raw or insufficiently cooked meat that contains *T. spiralis* muscle larvae (ML). The impact of *T. spiralis* on human health is enormous, with 65 818 people having been infected and 42 people having died from 1986 to 2009 worldwide [2]. Trichinellosis is a public health concern that also causes significant economic losses in the breeding pig industry and in terms of animal food safety [3]. Currently, albendazole is the first choice for the treatment of trichinellosis, but no commercially available vaccines are available. Clinical diagnosis is difficult due to the lack of specific clinical manifestations and effective early diagnostic antigens, making the control of trichinellosis challenging [4–6]. Therefore, to develop therapeutic or diagnostic strategies for trichinellosis, it is important to fully understand the molecular characteristics of ES proteins.

*Trichinella spiralis* has a simple life cycle with four developmental stages completed within a single host: ML, intestinal infective larvae (IIL), preadult (PA), adult (Ad) worms, and newborn larvae (NBL) [7]. When the host ingests infected muscle tissue, ML are released from their encapsulation by digestive enzymes in the stomach and develop into IIL at 0.9 h post-infection (hpi) [8]. Upon activation by duodenum juice, all IIL invade the intracellular niche of the small intestine epithelium within 6 hpi, where they develop into Ad through four moults at 30 hpi [8–10]. On Day 3 post-infection, the development and mating of Ad worms has been completed, and NBL are released within 5–10 days post-infection (dpi). NBL invade striated muscle cells through the circulatory system and lymphatic system and develop into infectious ML encapsulated within 3–4 weeks [11, 12] to complete the life cycle of *T. spiralis*. All the stages, ML, IIL, PA 6 h, PA 30 h, Ad 3 dpi and NBL, are essential for the survival and development of *T. spiralis* [13]. ES products from different stages are directly exposed to the immune system of the host and are interconnected messengers in the host-parasite relationship.

ML-ES products are the most commonly used serodiagnostic antigens recommended by the International Commission on Trichinellosis (ICT) because they are easily collected by cultivated ML in vitro and have considerable sensitivity and specificity [5]. However, there is an obvious difference between *T. spiralis* infection and antibody positivity during the early stages [14, 15]. This may be because stage-specific antibodies produced by intestinal-phase worms cannot recognize ML-ES antigens produced 3–4 weeks after infection [16]. Previous studies have shown that when ES antigens from 6 h IIL were used for the detection of anti-*T. spiralis* IgG antibodies, positivity of infected mouse sera could be detected as early as 10 dpi, with a higher sensitivity (100%) and specificity (96.86%) than ML-ES [17]. Using the ES antigens of Ad allowed the detection of IgG antibodies in infected mice as early as 8 dpi, with a sensitivity (100%) and specificity (98.11%) that were also higher than those of ML-ES [15]. The recombinant L20h-Ts3 fusion protein was utilized in a serological test that had been previously reported to be effective at 7–14 dpi, during which the “blind window” period was reduced during the early stages of infection [18]. rTs31 and rTsSP obtained from ML-ES were also potential early diagnostic antigens [19, 20]. In addition, rTs-cystatin from IIL and rTs-NBLsp from the NBL stage were used as good vaccine candidates against *T. spiralis* [21, 22]. Therefore, studying secretory proteins at different developmental stages is highly important for the diagnosis and prevention of *T. spiralis* infection.

Currently, the development of proteomics methods, such as two-dimensional electrophoresis combined with Western blot analysis, MALDI-TOF/TOF–MS/MS, iTRAQ, and label-free quantitative mass spectrometry, has been beneficial for protein research. Label-free quantitative mass spectrometry proteomics, which has high sensitivity and a low false discovery rate, can detect variant proteins at different developmental stages and is important for studying host-parasite relationships. Currently, a wide variety of worm somatic proteins from different developmental stages, including ML, IIL, PA, Ad, and NBL, have been identified [10, 16, 23, 24]. ES proteins are exposed to hosts and play a key role in modulating the immune response to allow parasite survival and development [25]. Therefore, proteomic analysis of ES proteins from different developmental stages is necessary.

Three proteins have been identified in IIL ES through ES proteomic studies [26, 27]. In this study, label-free LC‒MS/MS combined with bioinformatics analysis of ES proteins from different developmental stages, namely, ML, IIL, PA 6 h, PA 30 h, Ad 3 dpi and Ad 6 dpi, was performed to explore key biological processes and specific proteins involved in invasion and survival. The aim of this study was to identify new molecular targets for the diagnosis and control of *T. spiralis* infection by analysing secretions that interact with the host.

## Materials and methods

### Parasites and animals

The *T. spiralis* strain used in this study (ISS534) was preserved in our laboratory by serial passage in female specific-pathogen-free (SPF) SD rats. ML were recovered by the standard pepsin-HCl digestion method from infected SD rats at 35 dpi [28]. To obtain IIL that did not invade the intramulticellular niche of the small intestine epithelium, the ML were activated with simulated duodenal juice at 37 °C in 5% CO_2_ for 2 h, as previously reported [29]. Then, the simulated duodenum juice was removed by washing the worms with PBS. Worms at PA 6 h, PA 30 h, Ad 3 dpi, and Ad 6 dpi (containing Ad and NBL worms) were collected from the intestines of infected rats according to previously described methods with some modifications [30]. Briefly, 120 SD rats (30 animals per group) were inoculated with 10 000 ML and sacrificed at the desired timepoints of different worm development stages. The intestines were excised, and the contents of the intestine were removed by gently washing with PBS. Then, the intestines were placed on gauze in PBS at 37 °C for 1 h. The sediment at the bottom was recovered from the worms by washing 3 times with PBS.

### Extraction of ES proteins during different developmental stages

The ES proteins from ML, IIL, PA 6 h, PA 30 h, Ad 3 dpi, and Ad 6 dpi (containing Ad and NBL) were prepared as previously described [30–32]. First, the parasites were collected as described above, washed with PBS supplemented with antibiotics (100 U/mL penicillin, 100 μg/mL streptomycin) (Gibco, USA) 3 times and then washed in serum-free RPMI 1640 medium (Gibco, USA) supplemented with antibiotics 3 times. Then, the ML, IIL, PA 6 h, PA 30 h, Ad 3 dpi, and Ad 6 dpi parasites were incubated in prewarmed serum-free RPMI 1640 medium supplemented with antibiotics at 37 °C for 18 h under 5% CO_2_. The incubation density of ML, IIL, PA 6 h, and PA 30 h was 5000 worms/mL, and that of Ad 3 dpi and Ad 6 dpi was 50 worms/mL. After incubation, the supernatant was collected, and the parasites were discarded by centrifugation at 1000 × *g* for 5 min. The supernatant containing the ES proteins was filtered through a 0.22 μm membrane (Millipore, USA) and concentrated to 500 μL using Ultra15 3 kDa centrifugal filters (Millipore, USA) via repeated centrifugation at 3500 × *g* for 30 min, after which the mixture was centrifuged 5 times with PBS until the solution became colourless. The protein content in the ES samples at different stages was determined by the bicinchoninic acid (BCA) (Beyotime, China) method according to the kit manufacturer’s instructions, after which the samples were stored at −80 °C until use. In addition, 12% SDS‒PAGE gels stained with Coomassie blue were used to analyse the expression of the ES proteins (12 μg/lane).

### Trypsin digestion

To digest the samples, 200 μg of each ES protein solution was reduced with 5 mM dithiothreitol (Sigma, USA) at 56 °C for 30 min and alkylated with 11 mM iodoacetamide (Sigma, USA) at RT for 15 min in the dark. Urea was dissolved in 50 mM Tris–HCl to a concentration of 1 M, and then, TEAB (Sigma, USA) was dissolved in 1 M urea to a concentration of 100 mM. Then, the samples were diluted by adding 100 mM TEAB dissolved in 1 M urea. Finally, the first digestion of the protein sample was performed by adding 4 μg of trypsin (Promega, USA) to the protein sample for incubation at 37 °C overnight, and the second digestion was performed by adding 2 μg of trypsin for incubation at 37 °C for 4 h. The processes of recovery, desalting and concentrating the peptides was performed as previously described [30].

### LC‒MS/MS analysis

The digested peptides were dissolved in LC–MS solvent A (0.1% (v/v) formic acid), and separation was performed using a nanoElute UPLC system. The LC elution gradient was set as follows: increase from 6 to 22% solvent B (0.1% formic acid in acetonitrile) over 44 min, then 22% to 35% B in 10 min and to 80% in 7 min; then, the mixture was held at 80% for the last 3 min. All steps were performed at a constant flow rate of 300 nl/min.

The peptides were subjected to ionization with a capillary ion source, and then MS analysis was performed with a times-TOF Pro instrument (Bruker, Germany). The applied electrospray voltage was 1.4 kV, and the precursor ions of the peptides and their fragments were detected and analysed via TOF. The MS scanning range was set as 100 to 1700 m/z. The data were collected using parallel accumulation serial fragmentation (PASEF) mode. One MS scan followed by 10 PASEF scans were used to collect the spectra. The charges of the precursor ions were in the range of 0–5, and the dynamic exclusion time of the tandem MS scan was set to 24 s to avoid repeated precursor ion scanning.

### Protein data analysis

The resulting LC–MS/MS data were processed using the MaxQuant search engine (version 1.6.6.0). The search parameters were as follows: the *T. spiralis* (18 572 sequences) database was obtained from the UniProt database and concatenated with the reverse decoy database to calculate the false discovery rate (FDR) caused by random matching. In addition, common contaminating bases were added to the database to eliminate the effects of contaminating proteins on the identification results. The enzyme cleavage method was trypsin/P, and up to 2 missing cleavages was allowed. The mass tolerance for precursor ions was set to 40 ppm in the first search and main search, and the mass tolerance for fragment ions was set to 0.02 Da. Carbamidomethyl on cysteine was set as a fixed modification, and oxidation on methionine and acetylation on the N-terminus of the protein were specified as variable modifications. The FDRs identified by protein identification and peptide spectrum matching (PSM) were adjusted to 1%.

### Bioinformatics analysis

The prediction of molecular functions and biological processes of differentially expressed ES proteins was performed by the Gene Ontology (GO), Kyoto Encyclopedia of Genes and Genomes (KEGG) [33], and Clusters of Orthologous Groups (COG) databases. The protein‒protein interactions of all differentially expressed proteins were determined via the STRING database (version 10.1), and all interactions with a confidence score > 0.7 (high confidence) were retrieved.

### Statistical analysis

Principal component analysis (PCA) was performed and the relative standard deviation (RSD) and Pearson’s correlation coefficient were calculated to analyse quantitative reproducibility of protein expression. The threshold for identifying differentially expressed proteins was a 1.5-fold change compared with one another, with a value of *P* < 0.05 considered to indicate statistical significance. The data were analysed using GraphPad Prism 6.0 software for Windows, and all the data are expressed as the mean ± standard error of the mean (SEM) of 3 independent experiments. Student’s *t* test was used to assess differences in the ES protein samples from different developmental stages.

## Results

### Analysis of differentially expressed ES proteins by SDS‒PAGE

**Figure 1:**
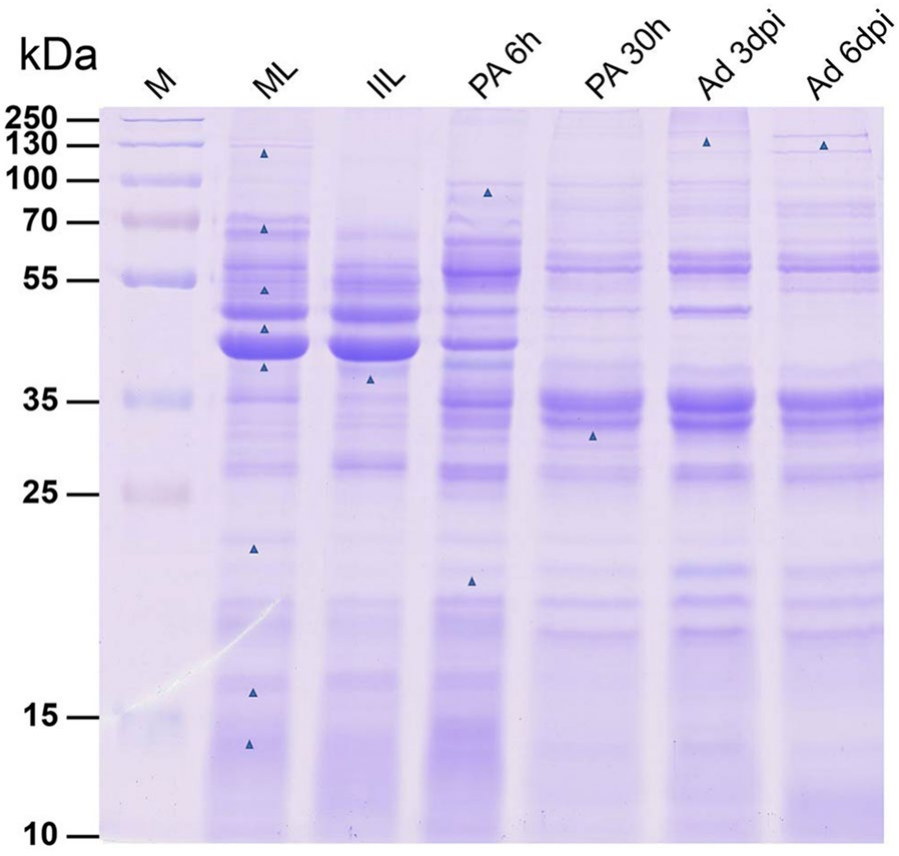
**SDS‒PAGE analysis of ES proteins at different developmental stages in *T. spiralis*.** ES proteins were separated on 12% polyacrylamide gels. Lane M: protein molecular weight marker; and Lanes 1–6: ES proteins of ML, IIL, PA 6 h, PA 30 h, Ad 3 dpi, and Ad 6 dpi, respectively. Triangles highlight divergent bands in the ES proteins during different developmental periods.

To identify stage-specific proteins, ES proteins were separated via SDS‒PAGE and stained with brilliant blue. As shown in Figure 1, the ES protein bands were clear and not degraded, and the molecular weights of the ES proteins from different developmental stages were between 10 and 190 kDa. The protein band with a molecular weight of 40 kDa was added to the other five developmental stages (Figure 1, lane IIL-Ad 6 dpi), and 21 kDa, 34 kDa, and 100 kDa proteins were added to PA 6 h-ES, PA 30 h-ES, Ad 3 dpi-ES, and Ad 6 dpi-ES (Figure 1, lane PA 6 h-Ad 6 dpi). The 190 kDa protein was added to Ad 3 dpi-ES and Ad 6 dpi-ES (Figure 1, lane Ad 3 dpi-Ad 6 dpi). The molecular weights of the missing protein bands in the other five developmental stages were 14 kDa, 17 kDa, 23 kDa, 43 kDa, 45 kDa, 50 kDa, 70 kDa, and 130 kDa (Figure 1, lane IIL-Ad 6 dpi). The specific protein bands were mainly observed between 20 and 70 kDa. The molecular weights of the expressed proteins were mainly between 40 and 50 kDa in ML-ES and IIL-ES (Figure 1, lane ML-IIL) [34, 35]. For the other four ES proteins, the molecular weights of the expressed proteins were mainly in the range of 25 to 35 kDa (Figure 1, lane PA 6 h-Ad 6 dpi) [19, 36].

### Identification and quantification of the differential expression profiles for the ES proteins using label-free analysis

**Figure 2:**
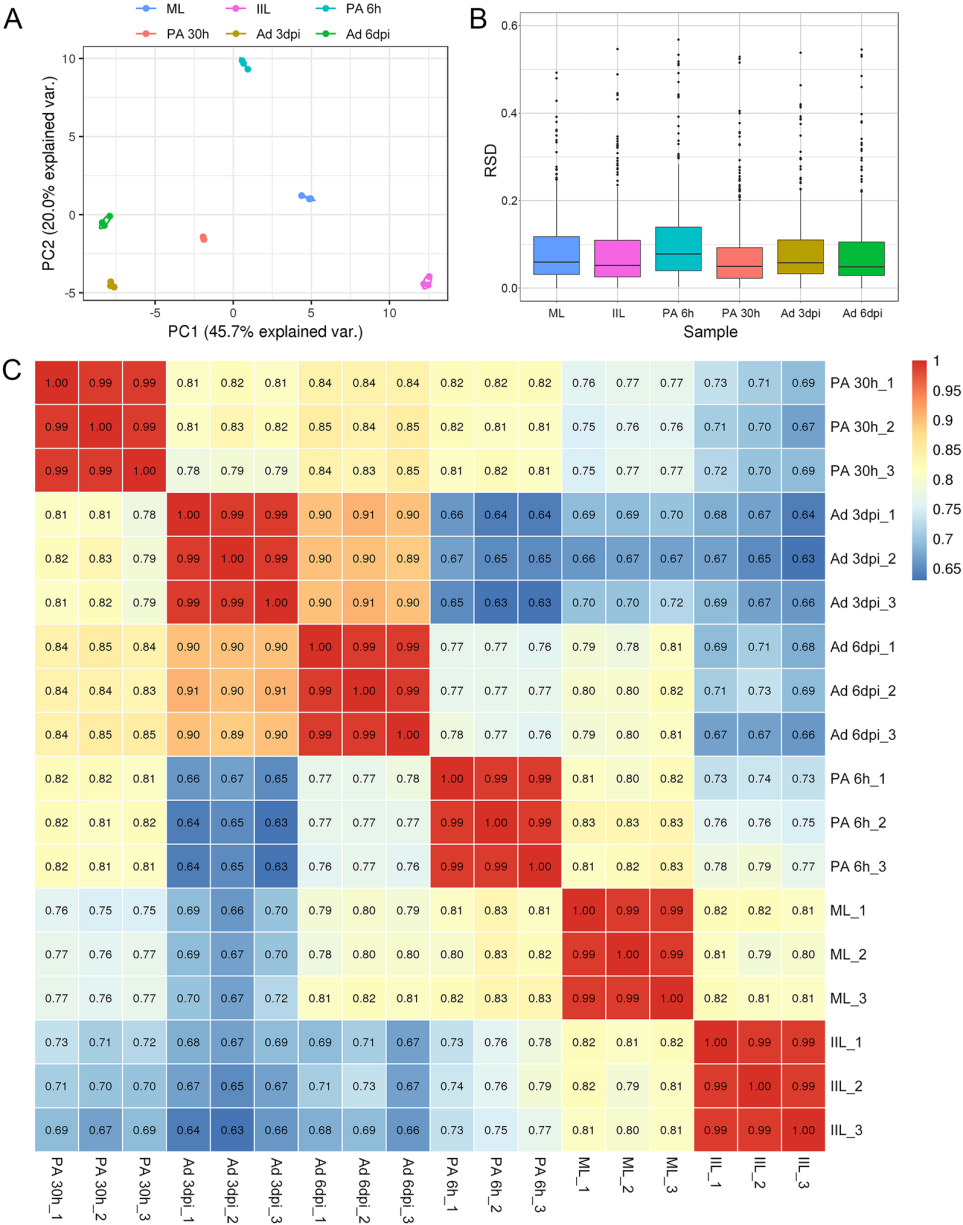
**Assessment of data quality.** **A** Principal component analysis. **B** Relative standard deviation analysis. **C** Pearson’s correlation coefficient analysis of secretory proteins from different developmental stages of *T. spiralis*. Three biological replicates were performed for each group.

A label-free quantitative mass spectrometry proteomics analysis was performed to study the differentially expressed ES proteins from different developmental stages of *T. spiralis*. Each ES protein consisted of three biological replicates, and pairwise comparisons were used to analyse the differentially expressed proteins from the six developmental stages. To assess the reproducibility of the data, we performed PCA and calculated the RSD and Pearson correlation coefficient to evaluate the quantitative reproducibility of the data [37]. The results in Figure 2A revealed that 65.7% of the variance explained by the PCA clustered tightly between repeated samples. Significant differences were found between groups. The overall RSD was less than 0.15 (Figure 2B). The Pearson correlation coefficient between repeated samples was strongly positive, and the r value was close to 1 (Figure 2C).

**Figure 3:**
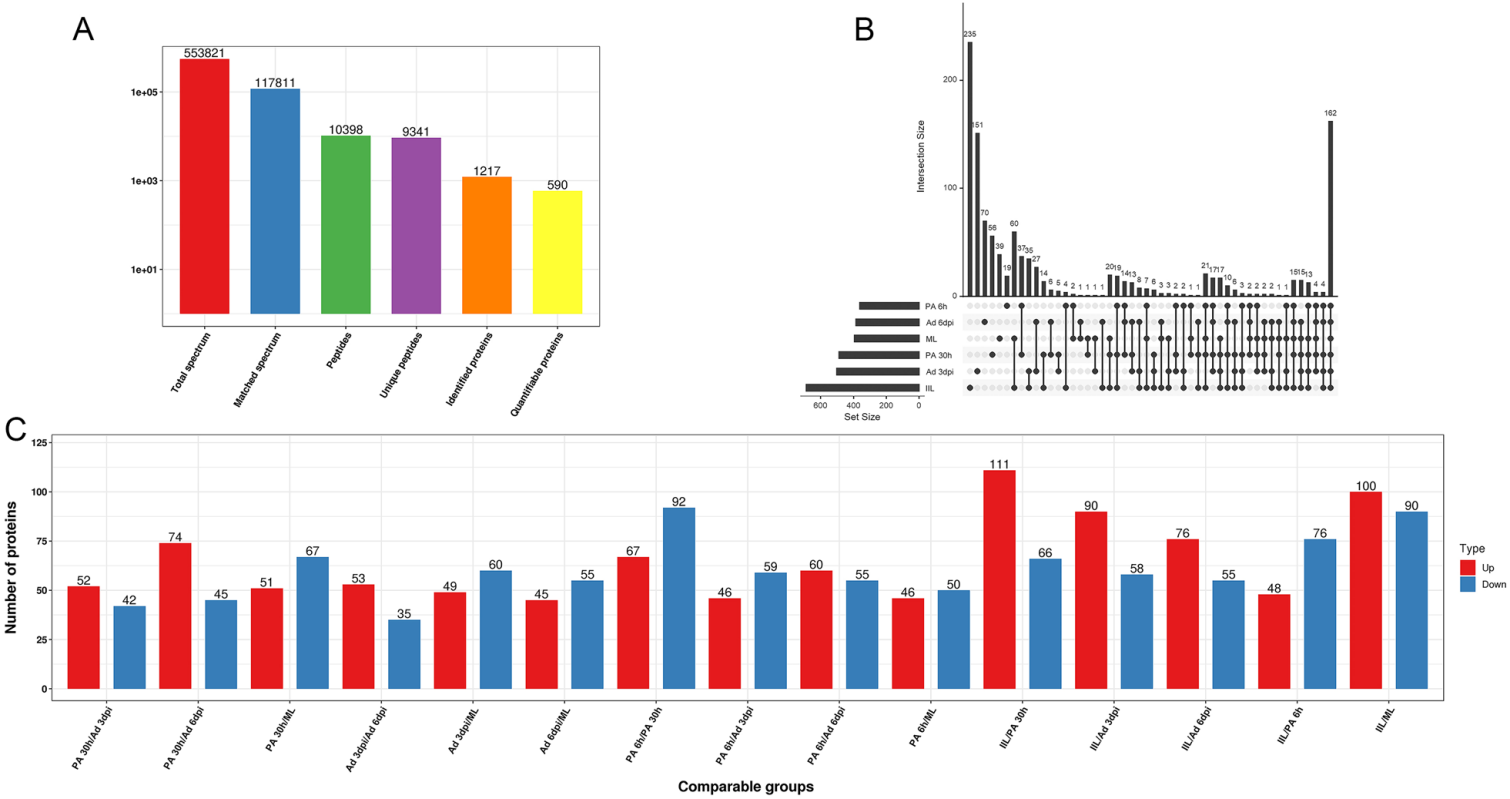
**Label-free analysis of proteins in *T. spiralis* at different developmental stages*.*** **A** LC‒MS/MS spectra identification and quantitative information. **B** Venn diagram showing the stage-specific and commonly expressed proteins. **C** Distribution of differentially expressed proteins filtered with a fold change threshold > 1.5 and a *P* value < 0.05.

Proteomic analysis revealed a total of 553 821 spectra (including 117 811 available matched spectra), 10 398 peptides (including 9341 unique peptides), and 1217 proteins from six different developmental stage ES proteins, 590 of which were differentially expressed and quantifiable proteins identified by pairwise comparisons of the two arbitrary stages (Figure 3A). Among the 1217 identified proteins, 395, 688, 361, 489, 501, and 384 proteins were identified in the ML-ES, IIL-ES, PA 6 h-ES, PA 30 h ES, Ad 3 dpi-ES, and Ad 6 dpi-ES groups, respectively. Of these proteins, 39, 235, 19, 56, 151, and 70 were stage-specific proteins in ML-ES, IIL-ES, PA 6 h-ES, PA 30 h ES, Ad 3 dpi-ES, and Ad 6 dpi-ES, respectively. The number of stage-specific proteins was highest in IIL-ES and was 12-fold greater than that in PA 6 h-ES. In addition, 162 proteins coexisted and were commonly differentially expressed in all stages of *T. spiralis* (Figure 3B).

The ES proteins from different developmental stages were compared with the ML-ES proteins, as shown in Figure 3C. Among the 590 differentially expressed proteins, 100, 46, 51, 49, and 45 were significantly upregulated (*P* < 0.05), and 90, 50, 67, 60, and 55 were significantly downregulated (*P* < 0.05) in IIL-ES, PA 6 h-ES, PA 30 h-ES, Ad 3 dpi-ES, and Ad 6 dpi-ES, respectively (Figure 3C and Additional file 1). The most differentially expressed proteins are shown in Table 1. Mannose-6-phosphate isomerase, bm5834 isoform c, bm5834 isoform c, plancitoxin-1, and plancitoxin-1 were the most significantly upregulated proteins in IIL-ES, PA 6 h-ES, PA 30 h-ES, Ad 3 dpi-ES, and Ad 6 dpi-ES compared with ML-ES, respectively, and apple domain-containing protein, peptidase S1 domain-containing protein, serine protease 30, uncharacterized protein, and ubiquitin-protein ligase were the most significantly downregulated proteins, respectively. The protein bm5834 isoform c was the most commonly upregulated protein in PA 6 h-ES and PA 30 h-ES, while plancitoxin-1 was the most commonly upregulated protein in Ad 3 dpi-ES and Ad 6 dpi-ES (Table 1).

**Table 1:** **The significantly differentially expressed proteins in** ***T. spiralis*** **ES compared to ML-ES**

UniProt ID	Protein description	Gene	Stage	Ratio	*P* value
A0A0V1BCS6	Mannose-6-phosphate isomerase	T01_7294	IIL-ES	27.926 ↑	0.000000402
A0A0V1B360	Elongation factor 2	eef-2	IIL-ES	14.782 ↑	0.048676
A0A0V1BHU3	Putative splicing factor, arginine/serine-rich 7	rsp-7	IIL-ES	14.719 ↑	0.00000106
A0A0V1ASK3	Peptidylprolyl isomerase	Adsl	IIL-ES	14.389 ↑	0.010204
A0A0V1C1W5	Heat shock protein 83 (fragment)	Hsp83	IIL-ES	12.031 ↑	0.0000159
A0A0V1B1K9	Apple domain-containing protein (fragment) (predicted)	T01_10939	IIL-ES	0.12 ↓	0.000022
A0A0V1BYW3	PAN domain protein (predicted)	T01_5647	IIL-ES	0.125 ↓	0.00032152
A0A0V1BV17	Apple domain-containing protein (fragment)	T01_11687	IIL-ES	0.177 ↓	2.45E-08
A0A0V1BRP1	IgA FC receptor (predicted)	T01_2456	IIL-ES	0.199 ↓	0.0000183
A0A0V1AQK3	Transposon Ty3-G Gag-Pol polyprotein (predicted)	T01_811	IIL-ES	0.201 ↓	0.008878
A0A0V1B172	Bm5834, isoform c (predicted)	T01_1871	PA 6 h-ES	29.147 ↑	0.00124421
A0A0V1C2Y0	Bm2172 (predicted)	T01_9020	PA 6 h-ES	16.338 ↑	6.17E-08
E5S926	Poly(U)-specific endoribonuclease-like protein	K02A11.3	PA 6 h-ES	16.154 ↑	0.0028968
A0A0V1BB13	Antileukoproteinase	SLPI	PA 6 h-ES	12.765 ↑	0.000000851
A0A0V1BCS6	Mannose-6-phosphate isomerase	T01_7294	PA 6 h-ES	12.538 ↑	0.000000104
A0A0V1BNK0	Peptidase S1 domain-containing protein	T01_13872	PA 6 h-ES	0.085 ↓	0.0080758
A0A0V1BLS5	Uncharacterized protein	T01_3102	PA 6 h-ES	0.092 ↓	0.00022432
A0A0V1C075	Deoxyribonuclease-2-alpha (fragment)	DNase2	PA 6 h-ES	0.108 ↓	0.0003178
A0A0V1BBQ6	AA_TRNA_LIGASE_II_ALA domain-containing protein (predicted)	T01_12550	PA 6 h-ES	0.131 ↓	0.00000121
A0A0V1BUU1	F-box domain-containing protein	T01_1979	PA 6 h-ES	0.136 ↓	0.00000346
A0A0V1B172	Bm5834, isoform c (predicted)	T01_1871	PA 30 h-ES	58.864 ↑	0.00086
E5S926	Poly(U)-specific endoribonuclease-like protein	K02A11.3	PA 30 h-ES	58.562 ↑	0.0013583
A0A0V1BB13	Antileukoproteinase	SLPI	PA 30 h-ES	33.355 ↑	0.000000229
A0A0V1AV55	Deoxyribonuclease-2-alpha (predicted)	T01_3845	PA 30 h-ES	26.945 ↑	6.94E-09
A0A0V1BX66	Plancitoxin-1	T01_2159	PA 30 h-ES	26.254 ↑	0.0000811
A0A0V1BDE1	Serine protease 30	Prss30	PA 30 h-ES	0.009 ↓	0.00129711
A0A0V1C075	Deoxyribonuclease-2-alpha (fragment)	DNase2	PA 30 h-ES	0.014 ↓	5.77E-08
E5SUX9	Tropomyosin	T01_14125	PA 30 h-ES	0.041 ↓	0.000000604
A0A0V1B692	Myosin-4	unc-54	PA 30 h-ES	0.041 ↓	0.000000612
A0A0V1BLS5	Uncharacterized protein	T01_3102	PA 30 h-ES	0.086 ↓	0.0000161
A0A0V1AXA4	Plancitoxin-1	T01_8966	Ad 3 dpi-ES	109.106 ↑	0.00000119
A0A0V1BX66	Plancitoxin-1	T01_2159	Ad 3 dpi-ES	54.913 ↑	0.00107528
E5S926	Poly(U)-specific endoribonuclease-like protein	K02A11.3	Ad 3 dpi-ES	28.792 ↑	0.0020188
A0A0V1AV55	Deoxyribonuclease-2-alpha	T01_3845	Ad 3 dpi-ES	19.078 ↑	7.43E-09
A0A0V1C0K3	Conserved cysteine-glycine protein 2-like protein (predicted)	T01_14740	Ad 3 dpi-ES	16.062 ↑	0.0000169
A0A0V1B2X1	Uncharacterized protein	T01_5772	Ad 3 dpi-ES	0.004 ↓	0.0028613
A0A0V1BH58	Ubiquitin–protein ligase (predicted)	T01_8770	Ad 3 dpi-ES	0.02 ↓	0.00129505
A0A0V1BSV1	ADP-ribose pyrophosphatase, mitochondrial	Nudt9	Ad 3 dpi-ES	0.046 ↓	0.000000379
A0A0V1B506	Vitellogenin (fragment)	T01_5226	Ad 3 dpi-ES	0.049 ↓	0.0000163
A0A0V1B119	3-ketoacyl-CoA thiolase, mitochondrial (fragment)	ACAA2	Ad 3 dpi-ES	0.089 ↓	0.0120172
A0A0V1AXA4	Plancitoxin-1	T01_8966	Ad 6 dpi-ES	111.255 ↑	0.00041762
A0A0V1BX66	Plancitoxin-1	T01_2159	Ad 6 dpi-ES	30.127 ↑	0.0000166
A0A0V1C0K3	Conserved cysteine-glycine protein 2-like protein (predicted)	T01_14740	Ad 6 dpi-ES	15.415 ↑	0.0000173
A0A0V1AV55	Deoxyribonuclease-2-alpha (predicted)	T01_3845	Ad 6 dpi-ES	11.75 ↑	0.000000016
A0A0V1BSI0	Endoplasmic reticulum resident protein 44 (fragment)	ERP44	Ad 6 dpi-ES	11.23 ↑	0.0127971
A0A0V1BH58	Ubiquitin–protein ligase (predicted)	T01_8770	Ad 6 dpi-ES	0.02 ↓	0.0066583
A0A0V1C0Z6	Uncharacterized protein	T01_7410	Ad 6 dpi-ES	0.048 ↓	0.000000298
A0A0V1AJK3	U3 small nucleolar ribonucleoprotein IMP4 (predicted)	T01_12692	Ad 6 dpi-ES	0.064 ↓	0.0000822
A0A0V1BBQ6	AA_TRNA_LIGASE_II_ALA domain-containing protein (predicted)	T01_12550	Ad 6 dpi-ES	0.092 ↓	0.00000115
E5S422	OV-16 antigen	OV16	Ad 6 dpi-ES	0.112 ↓	0.0085047

↑: upregulated proteins; ↓: downregulated proteins.

The main stage-specific proteins in ML-ES were zinc finger protein-like 1-like protein, myosin regulatory light chain 1, cadherin-related tumour suppressor, and paramyosin. Eukaryotic translation initiation factor was the main stage-specific protein in IIL-ES. Pumilio domain-containing protein-like protein, RNA helicase, glycoprotein 3-alpha-l-fucosyltransferase A, and bis(5′-adenosyl)-triphosphatase enpp4 were the main stage-specific proteins in PA 6 h-ES. The main stage-specific proteins in the PA 30 h-ES group were the collagen alpha-5(VI) chain, putative cuticle collagen, cuticle collagen sqt-1, and cuticle collagen 6. Adult-specific DNase II, 60S ribosomal protein L, and the 26S proteasome non-ATPase regulatory subunit were the main stage-specific proteins in the Ad 3 dpi-ES group. Newborn larvae-specific DNase II-3, deoxyribonuclease-2-alpha, cuticlin-1, and cadherin-related hmr-1 were the main stage-specific proteins in the Ad 6 dpi-ES group (Table 2). The molecular functions of some of these stage-specific proteins are shown in Table 2.

**Table 2:** **Specific proteins involved in the different developmental stages of** ***T. spiralis***

UniProt ID	Protein description	Gene	Stage	Molecular function
A0A0V1BBI7	Zinc finger protein-like 1-like protein	Y45G12B.2	ML-ES	Cation binding; metal ion binding; protein binding; zinc ion binding
A0A0V1BEW5	Myosin regulatory light chain 1 (fragment)	mlc-1	ML-ES	Cation binding; metal ion binding; calcium ion binding; binding
A0A0V1BND4	Cadherin-related tumour suppressor	ft	ML-ES	Cation binding; metal ion binding; protein binding; calcium ion binding;
A3RLX8	Paramyosin	unc-15	ML-ES	Motor activity; pyrophosphatase activity; cytoskeletal protein binding; catalytic activity
A0A0V1AX65	Eukaryotic translation initiation factor 3 subunit G	eif3g	IIL-ES	Organic cyclic compound binding; heterocyclic compound binding; translation factor activity, RNA binding
A0A0V1AXY2	Eukaryotic translation initiation factor 3 subunit D	eif3d	IIL-ES	Organic cyclic compound binding; heterocyclic compound binding; translation factor activity, RNA binding
A0A0V1BHF6	Eukaryotic translation initiation factor 3 subunit J (fragment)	eif3j	IIL-ES	Organic cyclic compound binding; heterocyclic compound binding; RNA binding
A0A0V1BT21	Eukaryotic translation initiation factor 3 subunit K	EIF3K	IIL-ES	Organic cyclic compound binding; heterocyclic compound binding; ribosome binding
A0A0V1B7Y2	Pumilio domain-containing protein-like protein	T01_7684	PA 6 h-ES	Organic cyclic compound binding; heterocyclic compound binding; RNA binding
A0A0V1BCF7	RNA helicase (fragment)	pit	PA 6 h-ES	RNA binding; ATP binding; nucleoside phosphate binding; helicase activity
A0A0V1BVH3	Glycoprotein 3-alpha-L-fucosyltransferase A	FucTA	PA 6 h-ES	Fucosyltransferase activity; transferase activity, transferring glycosyl groups; transferase activity, transferring hexosyl groups
A0A0V1BYH2	Bis(5'-adenosyl)-triphosphatase enpp4	enpp4	PA 6 h-ES	Catalytic activity
A0A0V1B7B2	Collagen alpha-5(VI) chain	COL6A5	PA 30 h-ES	Structural constituent of cuticle; structural molecule activity
A0A0V1BGG1 A0A0V1BKK7 A0A0V1BY38	Putative cuticle collagen	col-155	PA 30 h-ES	Structural constituent of cuticle; structural molecule activity
A0A0V1BL19	Cuticle collagen sqt-1	sqt-1	PA 30 h-ES	Structural constituent of cuticle; structural molecule activity
E5SAG9	Cuticle collagen 6	rol-8	PA 30 h-ES	Structural constituent of cuticle; structural molecule activity
A0A0V0Z5H8	Adult-specific DNase II-2 (predicted)	T01_2763	Ad 3 dpi-ES	Deoxyribonuclease activity; catalytic activity; hydrolase activity, acting on ester bonds; nuclease activity
A0A0V1AKT0	Adult-specific DNase II-7 (predicted)	T01_12782	Ad 3 dpi-ES	Deoxyribonuclease activity; catalytic activity; hydrolase activity, acting on ester bonds; nuclease activity
A0A0V1AUT4 A0A0V1BIM9 A0A0V1BNA7 E5S528 E5SC25	60S ribosomal protein L	RpL	Ad 3 dpi-ES	Structural constituent of ribosome; structural molecule activity
A0A0V1BFA2 A0A0V1BFL4 A0A0V1BV37 E5SB28	26S proteasome non-ATPase regulatory subunit	Psmd	Ad 3 dpi-ES	Protein binding; binding
A0A0V0Z163	Newborn larvae-specific DNase II-3 (predicted)	T01_5211	Ad 6 dpi-ES	Deoxyribonuclease activity; catalytic activity; hydrolase activity, acting on ester bonds; nuclease activity
A0A0V1AQV4 A0A0V1AQX0 A0A0V1AS00 A0A0V1ASZ6 A0A0V1B080 A0A0V1B6V5 A0A0V1B6V8 A0A0V1B6X3	Deoxyribonuclease-2-alpha	DNase2	Ad 6 dpi-ES	Deoxyribonuclease activity; catalytic activity; hydrolase activity, acting on ester bonds; nuclease activity
A0A0V1ARS6 A0A0V1B2K1 A0A0V1B422 E5SLM5	Cuticlin-1	cut-1	Ad 6 dpi-ES	None predicted
A0A0V1AWF2 A0A0V1AWQ5	Cadherin-related hmr-1	hmr-1	Ad 6 dpi-ES	Cation binding; metal ion binding; protein binding; calcium ion binding

### Functional classification of the differentially expressed proteins using Gene Ontology

**Figure 4:**
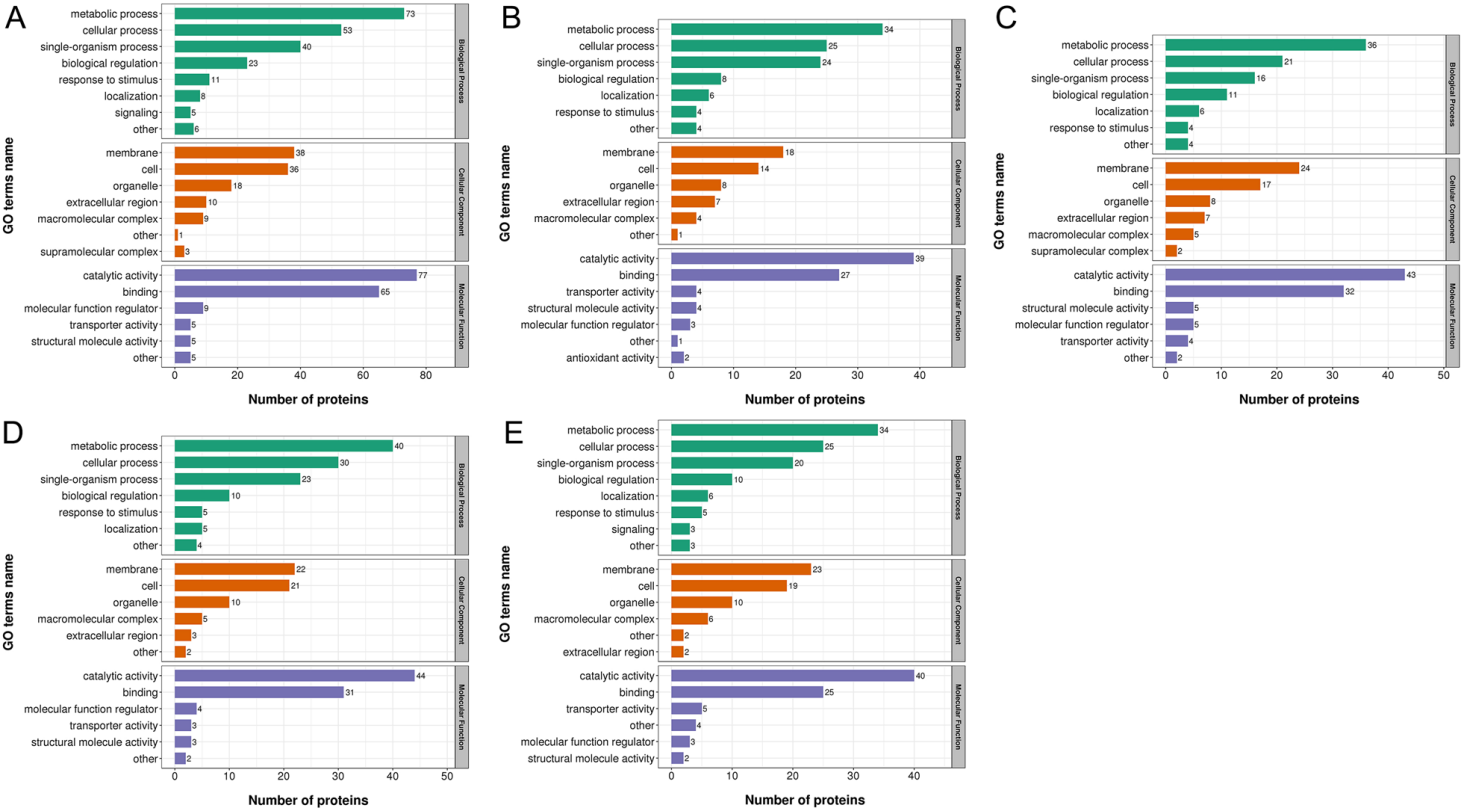
**Gene Ontology (GO) analysis of proteins in *T. spiralis* at different developmental stages*.*** **A** IIL vs. ML. **B** PA 6 h vs. ML. **C** PA 30 h vs. ML. **D** Ad 3 dpi vs. ML. **E** Ad 6 dpi vs. ML. The differentially expressed proteins were categorized into biological processes, cellular components, and molecular functions according to their GO annotation.

The biological functions of the differentially expressed proteins in all developmental stages of *T. spiralis* were predicted by GO classification analysis, which involved three categories, namely, biological process (BP), cellular component (CC), and molecular function (MF), based on their annotations in GO domains, which explained the biological functions of the differentially expressed proteins from different perspectives [38]. The results are shown in Figure 4. Compared with those in ML-ES, the main BPs associated with the differentially expressed proteins in IIL-ES were metabolic processes (73 proteins, 33%), cellular processes (53 proteins, 24%), and single-organism processes (40 proteins, 18%). According to CC, the differentially expressed proteins were distributed mainly in the membrane (38 proteins, 33%), cell (36 proteins, 31%), organelle (18 proteins, 16%), and extracellular region (10 proteins, 9%). According to MF, the main functions of the differentially expressed proteins were catalytic activity (77 proteins, 46%) and binding (65 proteins, 39%) (Figure 4A). For PA 6 h-ES, PA 30 h-ES, Ad 3 dpi-ES, and Ad 6 dpi-ES compared with ML-ES, the BP terms also predominantly included metabolic processes, cellular processes, and single-organism processes. The CC terms were also mainly associated with the membrane, cell, organelle, and extracellular region, except for those at 3 dpi-ES, and Ad 6 dpi-ES had a lower ratio of extracellular region. The main MFs of the differentially expressed proteins included catalytic activity and binding (Figures 4B–E).

### Subcellular localization of the differentially expressed proteins

**Figure 5:**
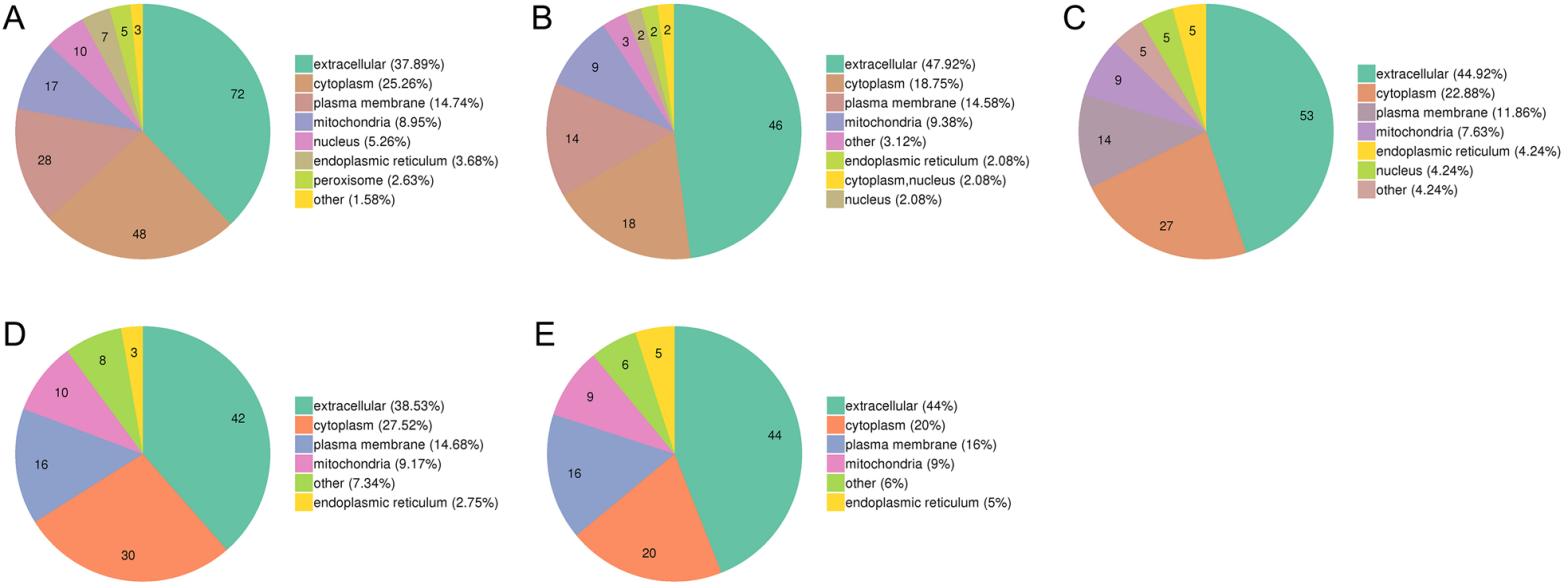
**Subcellular localization analysis of proteins in *T. spiralis* at different developmental stages*.*** **A** IIL vs. ML. **B** PA 6 h vs. ML. **C** PA 30 h vs. ML. **D** Ad 3 dpi vs. ML. **E** Ad 6 dpi vs. ML. The number in the pie chart denotes the number of proteins with the given subcellular localization annotation.

Subcellular localization of the differentially expressed proteins was analysed by WoLF PSORT software [39]. As shown in Figure 5, five different developmental stage ES proteins were compared with ML-ES, and the differentially expressed proteins were predominantly located in the extracellular space, cytoplasm, plasma membrane, and mitochondria.

### Clustering of orthologous groups (COG) analysis

**Figure 6:**
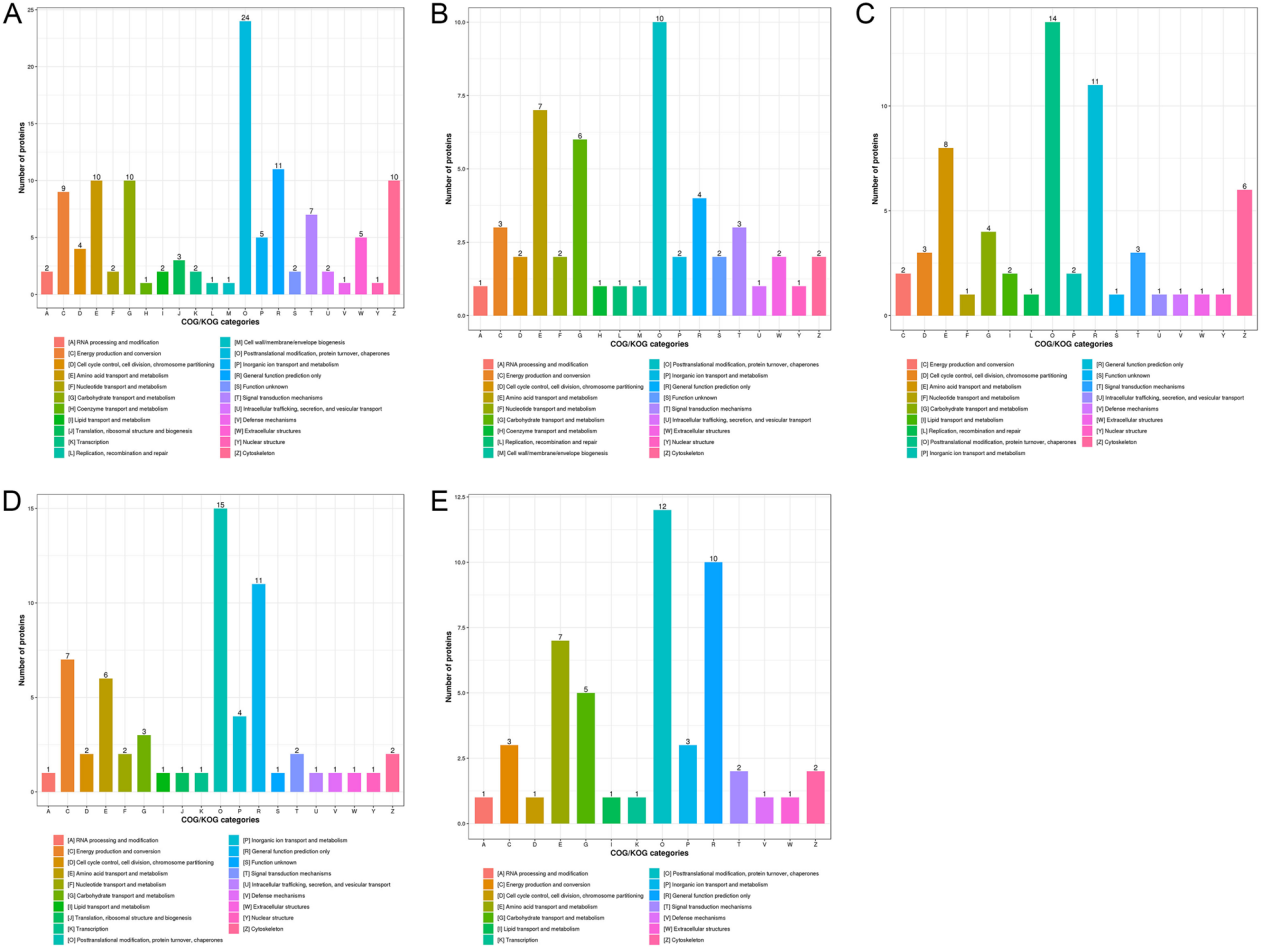
**Cluster of Orthologous Groups (COG) analysis of proteins in *T. spiralis* at different developmental stages*.*** **A** IIL vs. ML. **B** PA 6 h vs. ML. **C** PA 30 h vs. ML. **D** Ad 3 dpi vs. ML. **E** Ad 6 dpi vs. ML. The x-axis indicates the functional classification, and the y-axis indicates the number of matched proteins.

All the differentially expressed proteins from the six developmental stages of *T. spiralis* were classified into 25 COG clusters. The results from ML-ES revealed that the differentially expressed proteins were primarily involved in post-translational modification, protein turnover, chaperones, signal transduction mechanisms, translation, ribosomal structure and biogenesis, cytoskeleton, carbohydrate transport and metabolism, amino acid transport and metabolism, intracellular trafficking, secretion, vesicular transport, and energy production and conversion [37]. Among these proteins, the largest proportion were proteins associated with post-translational modification, protein turnover, and chaperones (Figure 6).

### Kyoto encyclopedia of genes and genomes analysis

**Figure 7:**
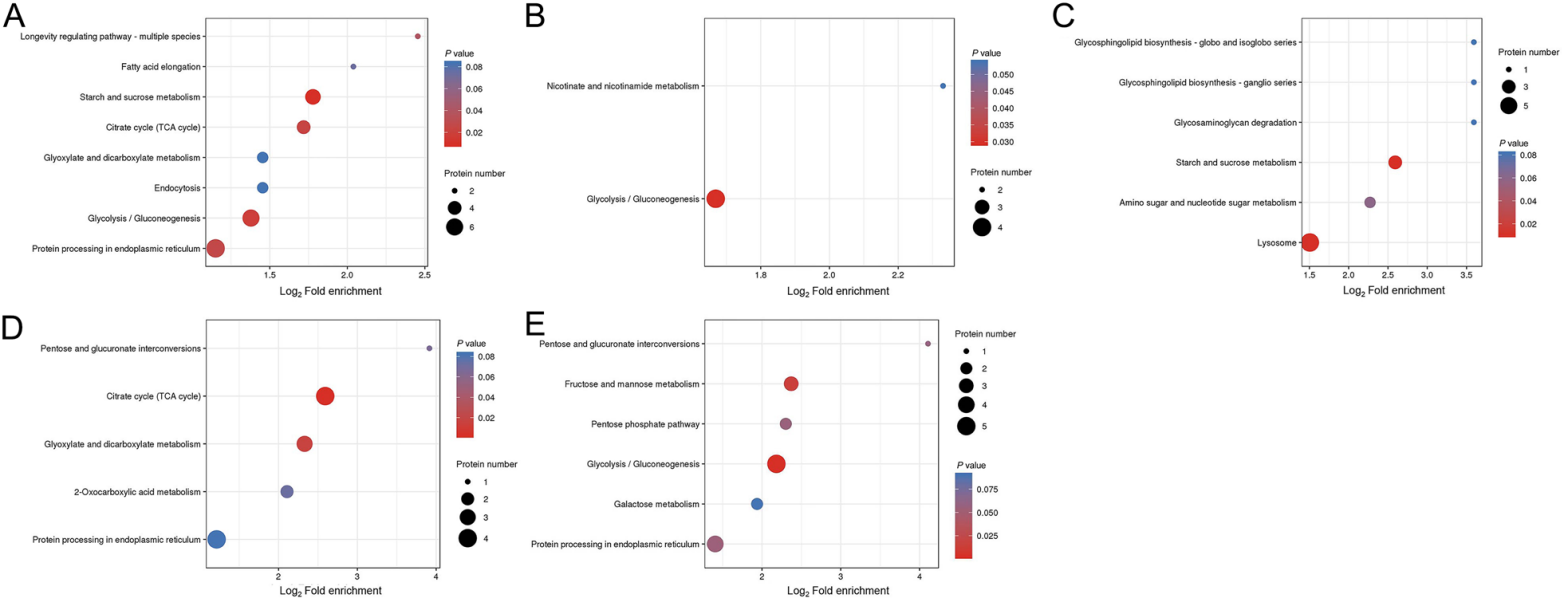
**Kyoto Encyclopedia of Genes and Genomes (KEGG) pathway enrichment analysis of proteins in *T. spiralis* at different developmental stages*.*** (A) IIL vs. ML. (B) PA 6 h vs. ML. (C) PA 30 h vs. ML. (D) Ad 3 dpi vs. ML. (E) Ad 6 dpi vs. ML. The x-axis indicates log_2_ (fold enrichment) values, and the y-axis indicates the KEGG pathway annotation.

**Figure 8:**
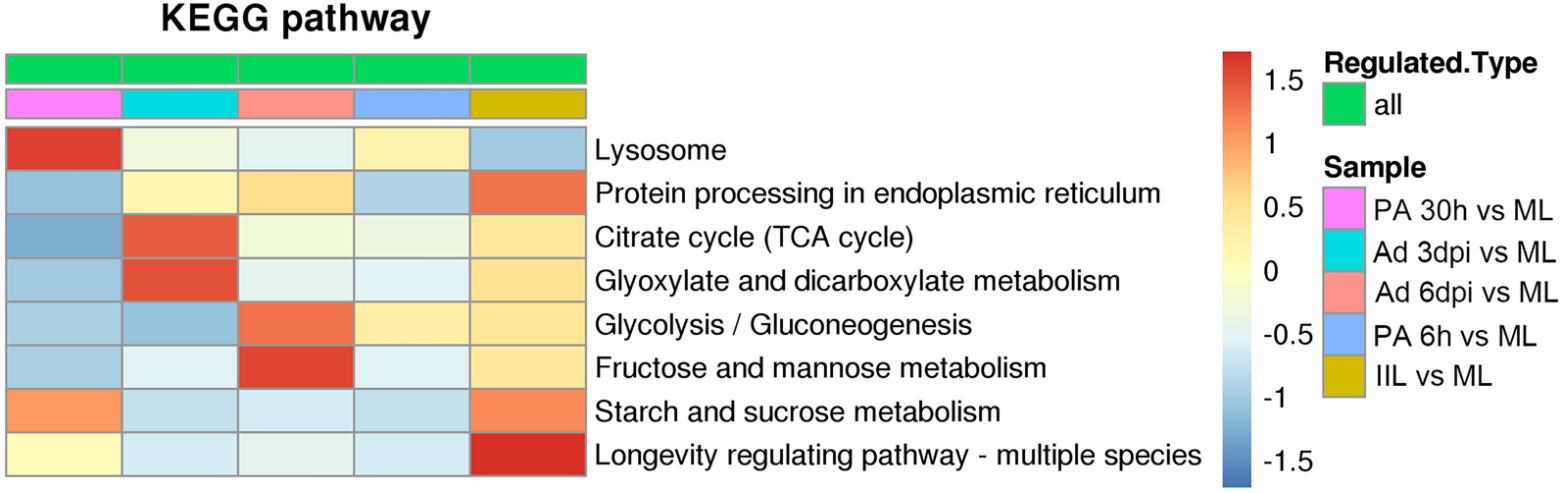
**Heatmap clustering of KEGG pathway enrichment analysis for the differentially expressed proteins.** **A** IIL vs. ML. **B** PA 6 h vs. ML. **C** PA 30 h vs. ML. **D** Ad 3 dpi vs. ML. (E) Ad 6 dpi vs. ML. The horizontal axis indicates the different comparison groups, and the vertical axis indicates the KEGG pathway annotation.

KEGG pathway analysis was performed to study the biological functions of the differentially expressed proteins in the six developmental stages [37]. These results are shown in Figure 7. Compared with those in ML-ES, the most significantly enriched pathways were involved in starch and sucrose metabolism, the citrate cycle (TCA cycle), glycolysis/gluconeogenesis, and protein processing in the endoplasmic reticulum in IIL-ES (Figure 7A); for PA 6 h-ES, only the glycolysis/gluconeogenesis pathways were enriched (Figure 7B); for PA 30 h-ES, starch and sucrose metabolism and lysosome pathways were enriched (Figure 7C); for Ad 3 dpi-ES, the TCA cycle and glyoxylate and dicarboxylate metabolism pathways were enriched (Figure 7D); and for Ad 6 dpi-ES, the enriched pathways were glycolysis/gluconeogenesis and fructose and mannose metabolism (Figure 7E). The results after heatmap clustering of enriched pathways were similar to the above results (Figure 8).

**Figure 9:**
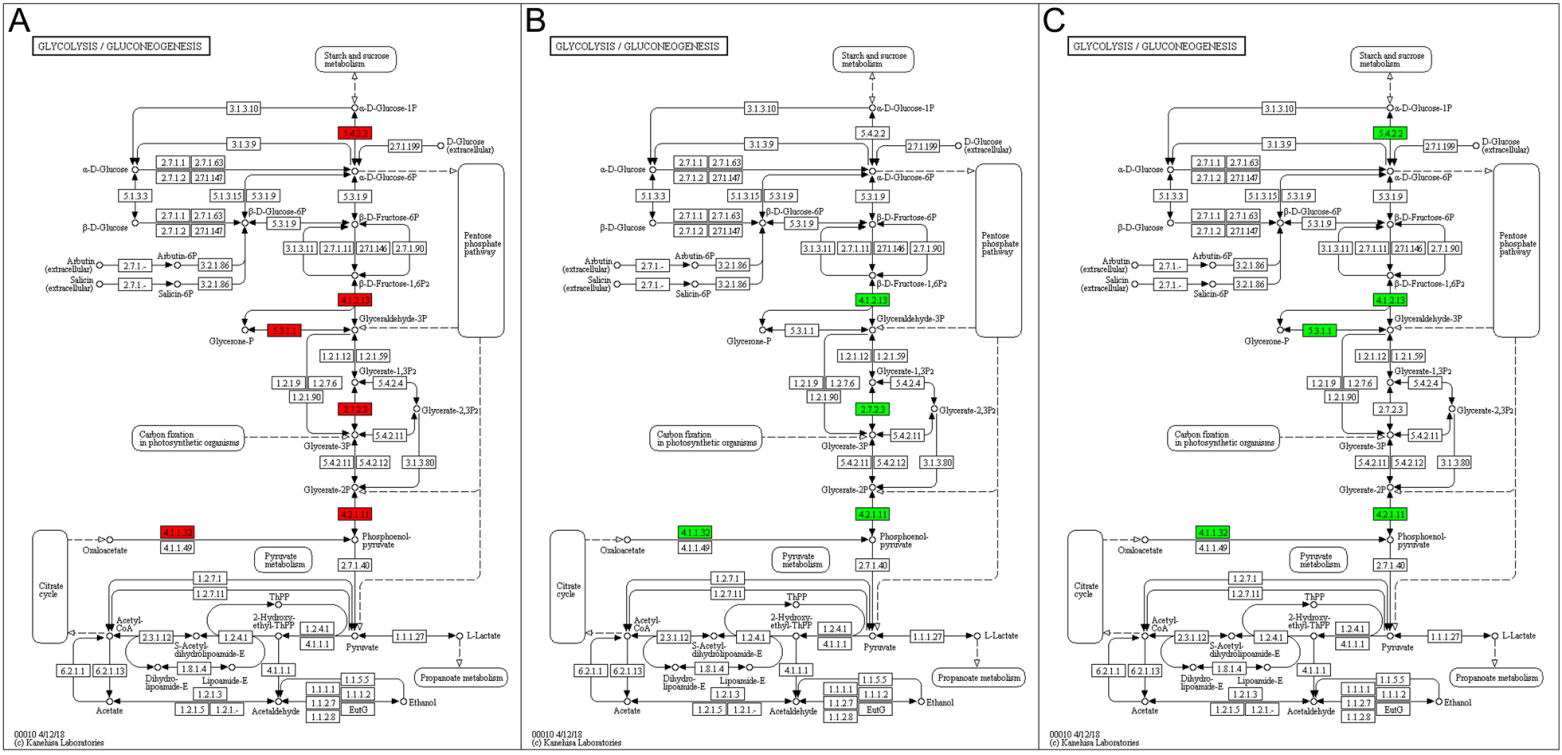
**KEGG pathway map of glycolysis/gluconeogenesis for the differentially expressed proteins.** **A** IIL vs. ML. **B** PA 6 h vs. ML. **C** Ad 6 dpi vs. ML. The red symbols represent upregulated proteins, and the green symbols represent downregulated proteins.

A pathway map of glycolysis/gluconeogenesis revealed that several related proteins, phosphoglucomutase (E5SQZ6), fructose-bisphosphate aldolase (A0A0V1AX39), triosephosphate isomerase (E5SS61), phosphoglycerate kinase (A0A0V1B8E2), enolase (A0A0V1BT87), and phosphoenolpyruvate carboxykinase (A0A0V1AZ55), were significantly upregulated in IIL-ES compared with ML-ES (*P* < 0.05) (Figure 9A). However, the proteins A0A0V1AX39, A0A0V1B8E2, A0A0V1BT87, and A0A0V1AZ55 were significantly downregulated in PA 6 h-ES compared with ML-ES (*P* < 0.05) (Figure 9B). For Ad 6 dpi-ES, the expression of the proteins E5SQZ6, A0A0V1AX39, E5SS61, A0A0V1BT87, and A0A0V1AZ55 was also significantly downregulated (*P* < 0.05) (Figure 9C). However, the glycolysis/gluconeogenesis pathway was not significantly different between PA 30 h-ES and Ad 3 dpi-ES. A pathway map of starch and sucrose metabolism revealed that, compared with those in ML-ES, E5SQZ6 and glycogen debranching enzyme (A0A0V1BQ16) were significantly upregulated and trehalase (A0A0V1BW15) was significantly downregulated in PA 30 h-ES (*P* < 0.05) (Additional file 2). A pathway map of the TCA cycle revealed that citrate synthase (E5SV78) and putative aconitate hydratase, mitochondrial (A0A0V1BRE7) were significantly upregulated, and malate dehydrogenase (E5SJF6) and A0A0V1AZ55 were significantly downregulated in Ad 3 dpi-ES compared to ML-ES (*P* < 0.05) (Additional file 3).

### Protein‒protein interaction (PPI) network analysis

**Figure 10:**
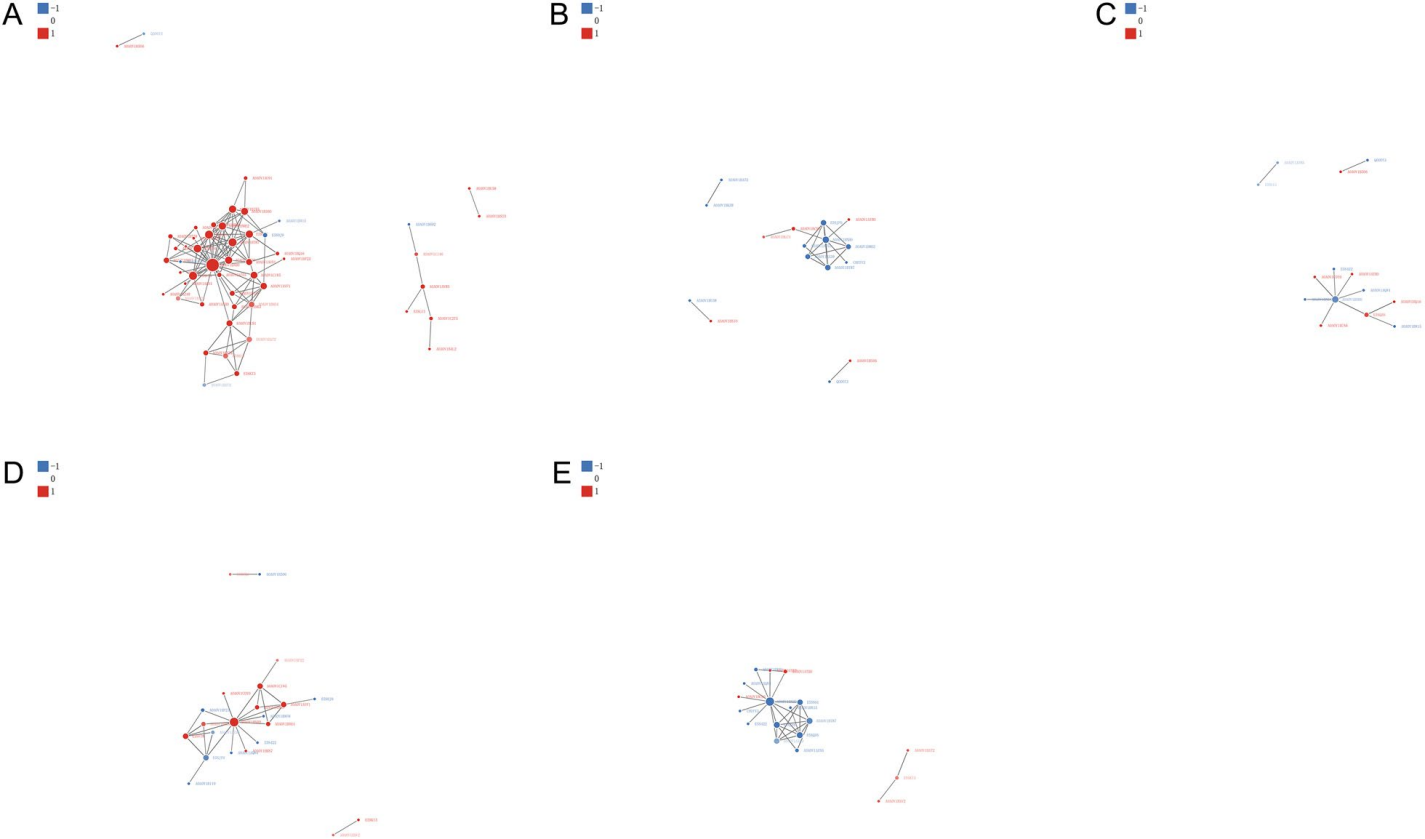
**Protein‒protein interaction (PPI) network analysis of the differentially expressed proteins.** **A** IIL vs. ML. **B** PA 6 h vs. ML. **C** PA 30 h vs. ML. **D** Ad 3 dpi vs. ML. **E** Ad 6 dpi vs. ML. The red symbols represent upregulated proteins, and the blue symbols represent downregulated proteins.

We performed a PPI network analysis for differentially expressed proteins in different developmental stages for comparison with ML-ES proteins using the STRING database (v. 10.5) [37]. To clearly demonstrate the PPIs, we selected the 50 proteins with the closest interactions. The results are shown in Figure 10, and compared with ML-ES, the upregulated heat shock cognate 71 kDa protein (A0A0V1BNH0) was the top interaction protein in the IIL-ES and Ad 3 dpi-ES (Figures 10A and D); however, the downregulated A0A0V1BNH0 protein was the top interaction protein in PA 6 h-ES, PA 6 h-ES, and Ad 6 dpi-ES (Figures 10B, C and E).

## Discussion

Trichinellosis is a zoonotic disease that seriously endangers human health and can cause damage to the myocardium and central nervous system [40]. *T. spiralis* has different developmental stages in a single host. Although the worm expulsion response occurs in the intestinal stage, NBL survival and successful parasitism are dependent on the parasite driving the immune system of the host towards a less dangerous response [41]. Secretory proteins in different developmental stages play key roles in regulating the immune responses of the host [41]. With advances in proteomics in recent years, it has become possible to investigate the proteomics of *T. spiralis* at different developmental stages. Proteomics can allow a better understanding of parasite biology and interactions between parasites and hosts. Investigating the proteomics of secretory proteins of *T. spiralis* is important for obtaining candidate vaccines and diagnostic antigens [42]. Several protein functions have been reported, such as 43-kDa/45-kDa and 35.5 kDa. These peptides can be used as antigens for the diagnosis of *T. spiralis* infection and as candidate vaccine molecules [19, 35, 36, 43]. Previous studies have focused on the worm somatic extract proteins of *T. spiralis* [10, 44]. However, secretory proteins have direct contact with the immune system of the host and play major roles in immune regulation [45, 46], and the secretory protein profiles from different developmental stages have not been characterized. Therefore, secretory proteins at different developmental stages were investigated using label-free analysis coupled with LC–MS/MS in this study.

In this study, we identified a total of 1217 proteins, 590 of which were differentially expressed according to pairwise comparisons of two arbitrary stages. Compared with those in ML-ES, our label-free proteomic analysis revealed 190, 96, 118, 109, and 100 differentially expressed proteins in IIL-ES, PA 6 h-ES, PA 30 h-ES, Ad 3 dpi-ES, and Ad 6 dpi-ES, respectively. As shown in Figure 3C, the differences between ML and Ad 6 dpi and between Ad 3 dpi and Ad 6 dpi were not obvious, even though there were fewer upregulated and downregulated proteins in Ad 6 dpi. We suggest that the differences at these two periods are due to the need to gestate newborn larvae because at 3 dpi, the nematodes are more mature and mating, whereas at Ad 6 dpi, most female nematodes had begun to produce newborn larvae [47]. The differentially expressed proteins were involved in metabolic processes, cellular processes, and single-organism processes; the membrane, cell, organelle, and extracellular region; and catalytic activity and binding. These differentially expressed proteins may play key roles in the evasion of host immune attack by parasites. The percentage of stage-specific proteins was close to 50%, indicating that the secretory proteins significantly differed among the different developmental stages. Stage-specific proteins cannot bind to early specific antibodies in adult worms, which are important for NBL and ML survival [48]. The label-free results were validated by PCA and the calculated RSD and Pearson’s correlation coefficient. The results obtained exhibited clear-cut strata clustering and high-quality data reproducibility, suggesting distinct differences in the different developmental stages and the reliability of our label-free analysis.

After infection, ML are released from encapsulation and emerge in the intestines of the host [8]. The IIL must adapt to this microenvironment and engage in various biological processes, such as invading the intestinal epithelial cells of hosts with environmental stimuli, which is the critical step for the successful parasitism of *T. spiralis* [24]. It has been reported that protein phosphorylation profiles change when ML are exposed to bile [49]. The protein profiles of the crude worm extract and ES also changed after ML were activated by bile, as determined by SDS–PAGE and Western blot analyses [50]. Based on the RT‒PCR results of a previous study, the expression of genes associated with bile-activated ML was altered compared to that associated with ML [24]. A previous study revealed that the surface proteins differed between ML and IIL, as determined by comparative proteomic analysis [44]. Our results revealed differences in secretory proteins between IIL and ML similar to those of the above study. However, comparison of the secretory proteins in the IIL stage versus the ML stage showed the most significant upregulation of mannose-6-phosphate isomerase (T01_7294), which is an essential enzyme in the metabolic pathways in parasites and is involved in the GDP-alpha-d-mannose biosynthetic pathway [51, 52]. Apple domain-containing protein is important for *Toxoplasma gondii* invasion [53]. However, the expression of apple domain-containing protein (T01_10939) was more significantly downregulated in IIL than in ML. It is possible that apple domain-containing protein plays a key role in the process of ML invasion of striated muscle cells.

In the intestinal stage, the parasite moults, develops between PA 6 h and PA 30 h and is initially exposed to the intestinal epithelium of the host, which may stimulate the immune response [23]. In our study, the proteins bm5834, isoform c, poly(U)-specific endoribonuclease-like protein, and antileukoproteinase were commonly significantly upregulated, and DNase2 was commonly significantly downregulated in both the PA 6 h and PA 30 h stages compared with the ML stage. Bm5834 isoform c was an uncharacterized protein. Poly(U)-specific endoribonuclease-like protein (also upregulated at Ad 3 dpi), with poly(U)-specific endoribonuclease activity, may participate in the splicing of heterogeneous nuclear RNA (hnRNA) [54]. Antileukoproteinase, also known as secretory leukocyte peptidase inhibitor (SLPI), acts as a local tissue protease inhibitor with limited systemic expression and protects the mucosal epithelia against inflammatory damage [55]. A previous study reported that DNase2 was the most abundant protein in the ML stages of *Trichinella* *britovi* and plays a key role in the parasite’s invasion, development and survival [56].

In the adult worm stage, to adapt to the local environment, parasites can express different proteins, which can regulate the immune response of the host [57, 58]. In this study, there were more differentially expressed proteins in the Ad 3 dpi group than in the Ad 6 dpi group, which was consistent with the findings of a previous study [16]. Similarly, compared with the ML group, the expression of plancitoxin-1 and deoxyribonuclease-2-alpha was significantly upregulated in the PA 30 h, Ad 3 dpi, and Ad 6 dpi groups. Ubiquitin-protein ligase was commonly significantly downregulated in both the Ad 3 dpi and Ad 6 dpi groups compared with the ML group. Plancitoxin-1 and deoxyribonuclease-2-alpha are members of the DNase II-like protein family and are able to catalyse the cleavage of DNA molecules into oligonucleotides [59]. Our previous study showed that plancitoxin-1 is a somatic protein distributed throughout NBL and the tegument of Ad and ML. Moreover, the recombinant plancitoxin-1 protein expressed via a prokaryotic expression system has nuclease activity [60]. Ubiquitin-protein ligases are distributed in secretory products, stored in the secretory organ of *T. spiralis* in the ML stage and participate in modification of the skeletal muscle of the host [61]. According to the results here, some proteins were present only in the larval stage. These proteins may be secreted during the larval stage and may help larvae invade and parasitize muscle cells.

Stage-specific secretory proteins at different developmental stages are important for parasitic evasion of attack due to the host immune response and survival [46]. According to our results, myosin regulatory light chain 1, paramyosin and cadherin-related tumour suppressors were found to be stage-specific proteins in ML. Myosin regulatory light chain 1 is a member of a large family of contractile proteins associated with the contraction of the smooth muscle of the parasite [62]. The recombinant myosin regulatory light chain elicited significant protection against infection with *Fasciola hepatica* [63]. Paramyosin has been identified in the ML stages of *T. spiralis* and is associated with the structure and motor activity of *T. spiralis* [56, 64]. The cadherin-related tumour suppressor protein has a tumour suppressor function in human hepatocellular carcinoma [65]. Eukaryotic translation initiation Factor 3 is a stage-specific protein in IIL that is involved in host cell invasion and is required for the growth of *Eimeria tenella* [66]. Among the stage-specific proteins in PA 6 h, both pumilio domain-containing protein-like protein and RNA helicase are involved in the process of RNA binding, while RNA helicase participates in regulating distinct steps of mRNA and preribosomal RNA metabolism [67]. However, our previous study revealed that RNA helicase was expressed in *T. spiralis* Ad 3 dpi [68]. This may be because our previous study results were generated from the Ad 3 dpi cDNA library, which indicated the total RNA transcription level. In this study, we analysed secretory protein levels at different developmental stages. Among the stage-specific proteins detected in PA 30 h, collagen alpha-5(VI) chain, putative cuticle collagen, cuticle collagen sqt-1, and cuticle collagen 6 are involved in the structural constitution of the cuticle and are the moulting-related proteins of *T. spiralis* that have been reported as crude PA 10 h antigens [10]. In this study, we demonstrated that moulting-related proteins also exist among the secretory proteins of *T. spiralis*, mainly at the PA 30 h stage. DNase II homologues play key roles in the development and homeostasis of *C. elegans* [69]. In a previous study, adult-specific DNase II proteins were identified in the ES from Ad 3 dpi via immunoproteomic analysis of early infection sera, and these proteins were shown to induce protective immunity during *T. spiralis* infection [42, 70, 71]. Our results also indicated that the adult-specific DNase II proteins existed only in Ad 3 dpi. The DNase II proteins were also the main specific proteins in Ad 6 dpi. Therefore, we speculated that DNase II proteins are important for the growth and development of *T. spiralis* adults and NBL. In addition, the cuticlin-1 protein, which is a noncollagenous cuticular protein that is expressed in the dauer larval stage of *C. elegans*, was abundant in Ad 6 dpi [72]. The protein cuticlin-1 is the key component that induces the host immune response to invading parasites, has been identified in protein extracts of *T. spiralis* ML treated with exogenous nitric oxide and helps ML escape NO-mediated oxidative stress [73]. Parasite growth is accompanied by moulting, which causes the old cuticle to be discarded and replaced with a new one; thus, the protein cuticlin-1 may be an important component of the cuticle of NBL released from adult worms.

On the basis of our GO analysis, we found that a range of the differentially expressed proteins were involved in metabolic processes and cellular processes, membranes and cells, and catalytic activity and binding. All the related proteins involved in biological processes and functions may play key roles in *T. spiralis* growth and development, invasion of enterocytes, induction of the host immune response, and transformation of host cells into nurse cells and have structural roles as myosin complex proteins [23]. KEGG pathway analysis revealed that the differentially expressed proteins at different developmental stages were enriched mainly in energy metabolism pathways, which included glycolysis/gluconeogenesis, the TCA cycle, and starch and sucrose metabolism. The key enzymes phosphoglucomutase, fructose-bisphosphate aldolase, triosephosphate isomerase, phosphoglycerate kinase, enolase, and phosphoenolpyruvate carboxykinase, which are involved in the glycolysis/gluconeogenesis pathway, were significantly upregulated in the IIL stage compared to the other five stages, which indicates that glycolysis/gluconeogenesis is more active in the IIL stage. A plentiful supply of energy facilitates the motor activity of IIL and their invasion of enterocytes.

Proteins perform various functions by interacting with other proteins. To further explore the relationships between proteins differentially expressed at different developmental stages and ML-ES proteins, we used the STRING database to evaluate the PPIs. The results showed that the heat shock cognate 71 kDa protein was the centre of the interaction protein at different developmental stages. The heat shock cognate 71 kDa protein is located in the cytoplasm and is involved in metabolic processes, cellular processes, and single-organism processes; is associated with the MF terms binding and catalytic activity; and participates in the spliceosome pathway, the MAPK signalling pathway, protein processing in the endoplasmic reticulum, endocytosis, and longevity-regulating pathways in multiple species. Heat shock proteins (Hsps) are traditionally considered to be highly immunogenic proteins and are also potential diagnostic targets or vaccine candidates for the strong host immune response caused by parasites [12, 74–76]. Hsp70 is an immunogenic protein released by worms and exposed to the host immune system during infection, making it a possible candidate vaccine against *T. spiralis* [77]. Moreover, Hsp70 was recognized in early infection sera and may be applied in the early diagnosis of trichinellosis [12]. Hsp71 was obtained from heat shocked larvae, and its strong reactivity with a monoclonal antibody against Hsp70 indicates that it can elicit protective immunity, which renders the host more refractory to reinfection [78]. Therefore, we speculated that the heat shock cognate 71 kDa protein may play a key role in the development of *T. spiralis*, and its immunological protection and diagnostic capacity will be studied in the future.

In conclusion, label-free LC–MS/MS quantification was used in this study to explore ES proteins differentially expressed in different developmental stages of *T. spiralis*. A total of 590 proteins were found to be differentially expressed by pairwise comparison of ES proteins, and the BP, CC, and MF terms of these proteins were annotated. The differentially expressed proteins at different developmental stages were enriched mainly in the energy metabolism pathway. The common proteins (e.g., heat shock cognate 71 kDa protein) in different developmental stages of *T. spiralis* may be potential targets for parasitic diagnosis and vaccine candidates. Our study provides a valuable basis for the control of trichinellosis. Proteomic analysis of secretory proteins in *T. spiralis* at different developmental stages will help us to better understand the mechanisms by which this parasite regulates host immune responses.

### Supplementary Information

**Additional file 1. Differentially expressed proteins in different developmental stages of *T. spiralis*.** (A) IIL vs. ML. (B) PA 6 h vs. ML. (C) PA 30 h vs. ML. (D) Ad 3 dpi vs. ML. (E) Ad 6 dpi vs. ML. The x-axis indicates log_2_ (fold change) values, and the y-axis indicates –log_10_ (*P* value) values. The red dots represent upregulated proteins, and the blue dots represent downregulated proteins according to volcano plgots and compared to ML. The grey dots indicate that there are no significant differences.

**Additional file 2. Starch and sucrose metabolism-related genes associated with the differentially expressed proteins in *T. spiralis* PA 30 h.** The red symbols represent upregulated proteins, and the green symbols represent downregulated proteins compared to ML in the KEGG pathway map.

**Additional file 3. Differentially expressed proteins associated with the citrate cycle (TCA cycle) for the differentially expressed proteins in *T. spiralis* Ad 3 dpi.** The red symbols represent upregulated proteins, and the green symbols represent downregulated proteins compared to ML in the KEGG pathway map.

## Data Availability

All relevant data are within the manuscript and its supporting information files.
