# Supplementary figures and images for: Quantitative label-free proteomic analysis of excretory-secretory proteins in different developmental stages of Trichinella spiralis
Source: Vet Res. 2024 Jan 3;55:4. doi: 10.1186/s13567-023-01258-7 (PMC10763447; doi:10.1186/s13567-023-01258-7)

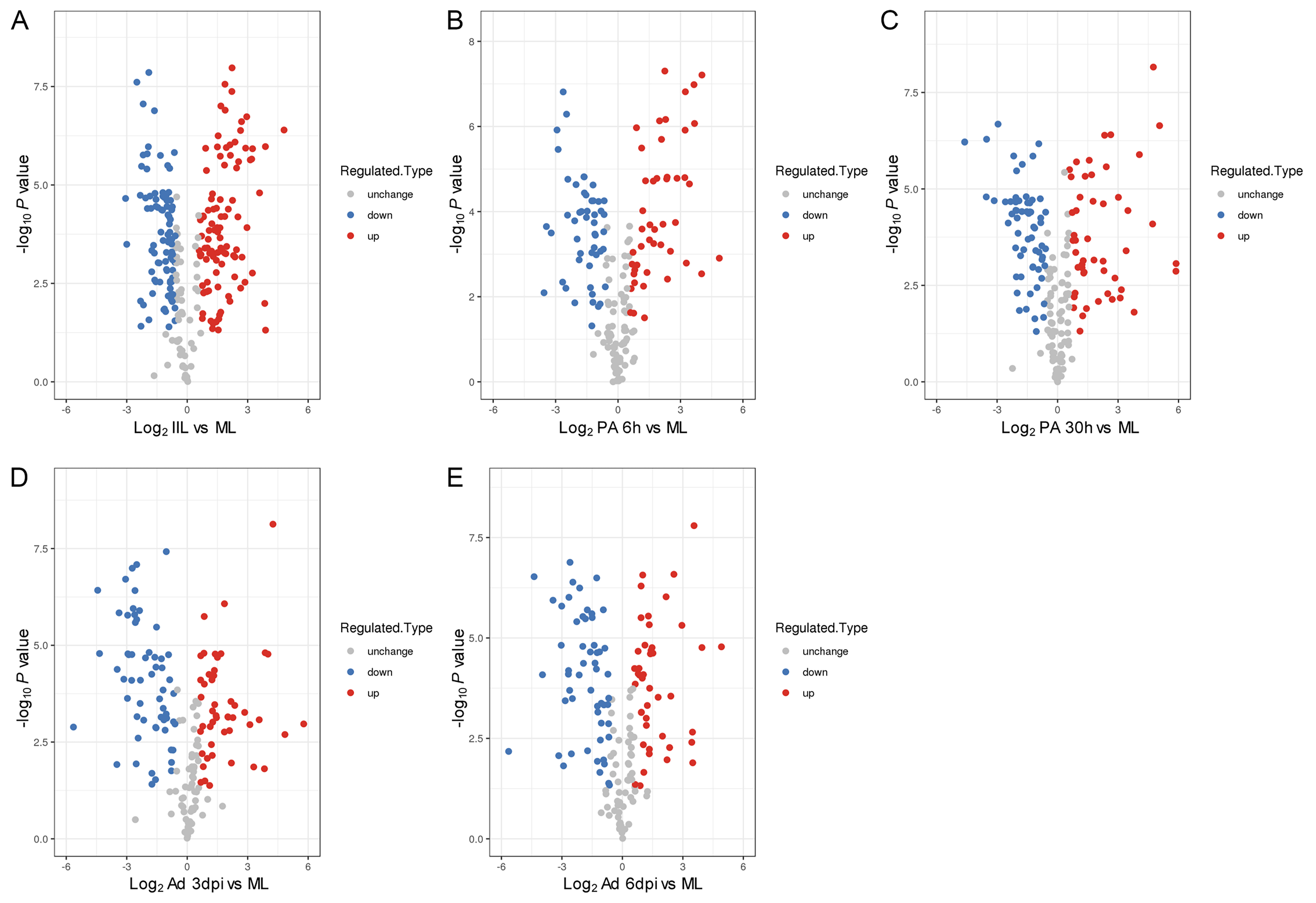

Supplement: Supplementary file 1 — Additional file 1. Differentially expressed proteins in different developmental stages of T. spiralis. (A) IIL vs. ML. (B) PA 6 h vs. ML. (C) PA 30 h vs. ML. (D) Ad 3 dpi vs. ML. (E) Ad 6 dpi vs. ML. The x-axis indicates log2 (fold change) values, and the y-axis indicates –log10 (P value) values. The red dots represent upregulated proteins, and the blue dots represent downregulated proteins according to volcano plgots and compared to ML. The grey dots indicate that there are no significant differences. [file 13567_2023_1258_MOESM1_ESM.jpg]

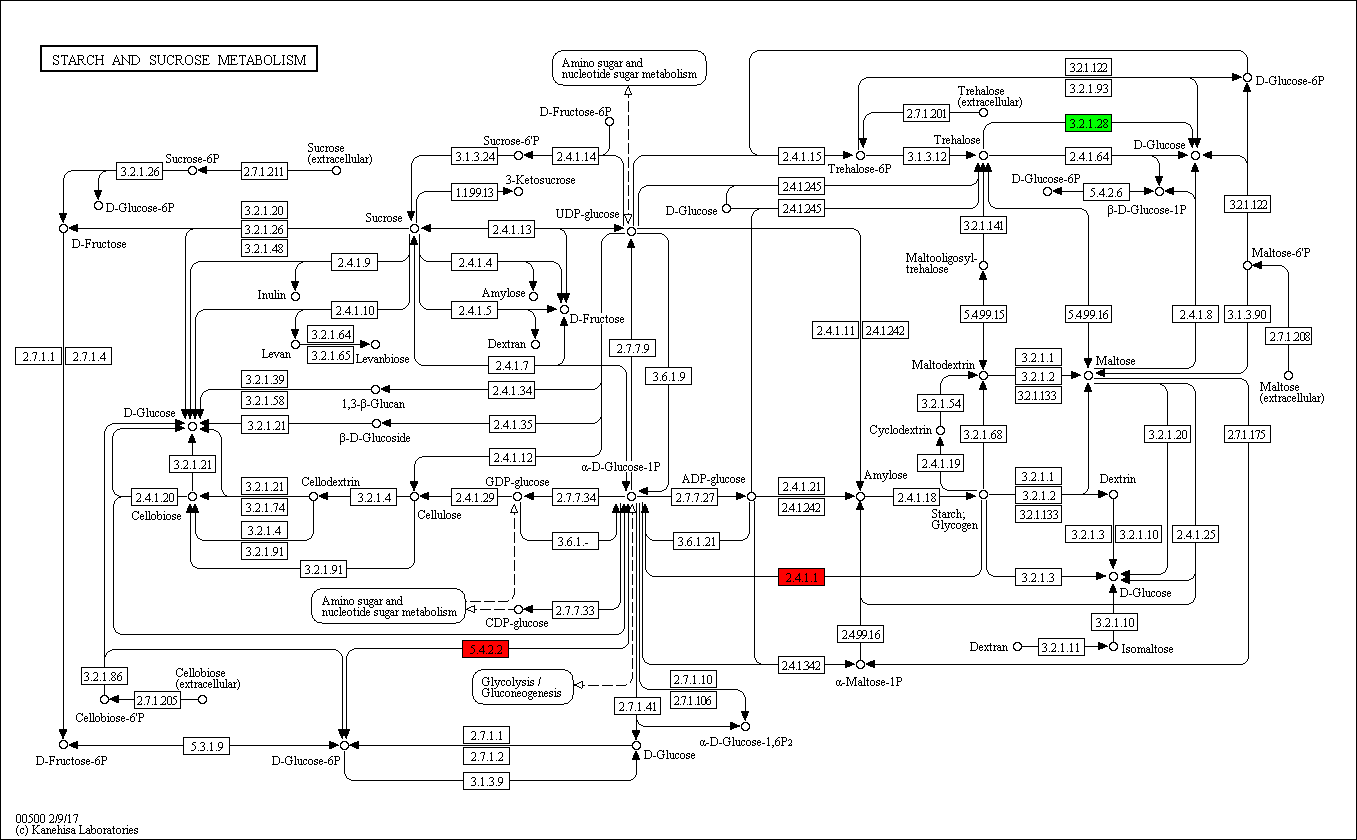

Supplement: Supplementary file 2 — Additional file 2. Starch and sucrose metabolism-related genes associated with the differentially expressed proteins in T. spiralis PA 30 h. The red symbols represent upregulated proteins, and the green symbols represent downregulated proteins compared to ML in the KEGG pathway map. [file 13567_2023_1258_MOESM2_ESM.png]

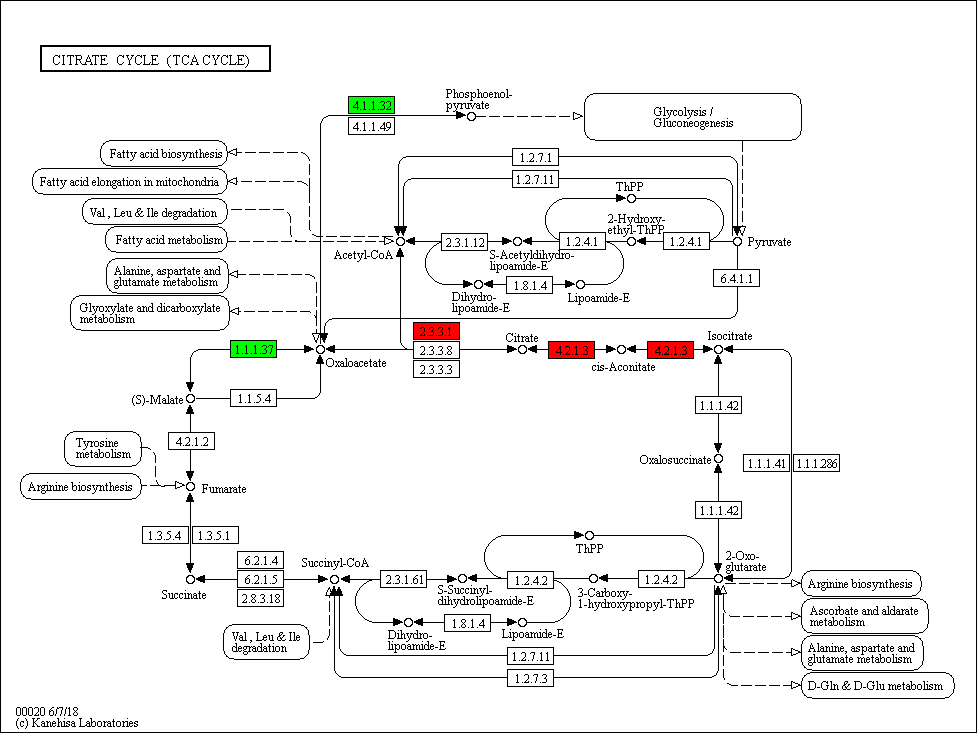

Supplement: Supplementary file 3 — Additional file 3. Differentially expressed proteins associated with the citrate cycle (TCA cycle) for the differentially expressed proteins in T. spiralis Ad 3 dpi. The red symbols represent upregulated proteins, and the green symbols represent downregulated proteins compared to ML in the KEGG pathway map. [file 13567_2023_1258_MOESM3_ESM.png]
